# New Molecular Approach for the Detection of Kinetoplastida Parasites of Medical and Veterinary Interest

**DOI:** 10.3390/microorganisms8030356

**Published:** 2020-03-02

**Authors:** Hacène Medkour, Marie Varloud, Bernard Davoust, Oleg Mediannikov

**Affiliations:** 1 IHU Méditerranée Infection - Microbes, Evolution, Phylogeny and Infection (MEΦI), 13385 Marseille CEDEX 05, France; hacenevet1990@yahoo.fr (H.M.); bernard.davoust@gmail.com (B.D.); 2 UMR Aix-Marseille Université, IRD, APHM -19-21, Bd Jean Moulin, 13385 Marseille CEDEX 05, France; 3 PADESCA Laboratory, Veterinary Science Institute, University Constantine 1, El Khroub 25100, Algeria; 4 CEVA Animal Health, 33500 Libourne, France; marie.varloud@ceva.com

**Keywords:** Kinetoplastida, diagnostic, qPCR, PCR, *Leishmania*, *Trypanosoma*

## Abstract

Kinetoplastids are protozoa containing a range of ubiquitous free_living species–pathogens of invertebrates, vertebrates and even some plants. Some of them are causative agents of canine vector-borne diseases. Their diagnosis is often missing in a gold standard. Here, we proposed a molecular approach for the diagnosis and study of Kinetoplastida. The TaqMan qPCR assays target the following genes: *24Sa LSU* of Kinetoplastida, *28S LSU* of *Leishmania/ Trypanosoma* spp., *5.8S* rRNA of *Trypanosoma* spp., *18S SSU* of *Leishmania* spp., kinetoplast minicircle DNA *(kDNA)* of *L. donovani* complex and *kDNA* of *L. infantum*, were designed, validated for their sensitivity (Se) and specificity (Sp) in silico and in vitro using a panel of known DNAs. They were then used to screen 369 blood samples (358 dogs, 2 equids, 9 monkeys). In addition, new *28S LSU* primer sets are presented to use for Kinetoplastida’s identification by PCR/sequencing. All qPCRs showed consistently high analytical sensitivities and reproducibility. They detect approximately 0.01 parasite/ mL blood for the *kDNA* based- qPCRs and at least a single cell-equivalent of rDNA for the other systems. Based on the sequencing results, after screening, Se and Sp were: 0. 919 and 0.971, 0.853 and 0.979, 1.00 and 0.987, 0.826 and 0.995 for all of Kinetoplastida, *Leishmania/ Trypanosoma*, *Trypanosoma*, *Leishmania* spp. specific qPCRs, respectively. kDNA based qPCRs were more sensitive and specific (Se: 1.00; Sp: 0.997). PCR/sequencing allowed the detection of Kinetoplastids in animal blood samples such as *L. infantum, L. guyanensis, T. congolense, T. evansi* and *Bodo* spp. The molecular approach proposed here is useful for epidemiological studies, fundamental research such as screening for new Kinetoplastida species, diagnosis and therapeutic follow-up. In addition, researchers are free to choose the molecular tools adapted to their aims.

## 1. Introduction

Kinetoplastida (or Kinetoplastea, as a class) are a group of flagellated protists belonging to the phylum Euglenozoa, characterized by the presence of a large massed DNA called kinetoplast (hence the name), an organelle that stains like the nucleus, but is actually a specific part of the mitochondrion in which large amounts of extranuclear DNA are accumulated [1,2]. Kinetoplastida contain a range of ubiquitous free-living species −pathogens of invertebrates, vertebrates and even some plants [3].

The order Trypanosomatida, whose members are exclusively parasites and which is mainly found in insects, is the most documented [4,5]. Trypanosomatidae is the only one Trypanosomatida family. Most monoxenic trypanosomatides with a single host parasitize insects. Dixenous parasites, with two hosts in their life cycle employ an invertebrate, arthropod or leech as a vector for transmission between vertebrate hosts, and are the genera *Endotrypanum*, *Leishmania*, *Paraleishmania*, *Trypanosoma* or plant genus *Phytomonas* hosts. However, the trypanosomatids were brought to prominence by two genera, *Trypanosoma* and *Leishmania*, attributable to their role as human and animal pathogens [6]. *Trypanosoma* and *Leishmania* are obligatorily dixenous, possess zoonotic or anthroponotic life-cycles and are transmitted by hematophagous insects [6,7]. These parasites cause devastating human diseases including human African trypanosomiasis due to *Trypanosoma brucei*, Chagas disease caused by *Trypanosoma cruzi* and the leishmaniases, which are attributable to about 20 species of *Leishmania* [8,9,10,11]. With the exception of the strictly anthroponotic *gambiense* form of human trypanosomiasis and infections caused by *Leishmania tropica* and *L. donovani*, for which humans are considered the main reservoir, diseases associated with trypanosomiasis are mainly zoonotic, with animal reservoirs playing a key role in maintaining endemicity [12,13,14]. This includes *L. infantum*, the causative agent of the most common vector-borne protozoan disease worldwide [15], *T. brucei* and *T. congolense*, the causative agents of Nagana or a similar disease in Africa and Asia, as well as *T. evansi*, the etiological agent of surra and the so-called “mal de cadeiras” outside Africa. Animals are also important reservoirs of *T. cruzi* in the Americas [16]

Much of the cellular biology of the different Kinetoplastids is very similar. Of the 8000 genes that constitute Kinetoplastids, more than 6000 are common orthologists, and the diseases they cause are very characteristic [17]. Understanding the differences between these disease–causing pathogens at genetic, molecular and cellular levels might provide new approaches to the development of diagnostics, vaccines and tools needed to control them [17].

The diagnosis of a range of pathogenic Kinetoplastids relies on clinical manifestations, epidemiological and laboratory data. With regard to laboratory methods, a gold standard for human or animal patients is often missing [18], which hinders the collection of accurate epidemiological data and thus limits disease control. In addition, false-negative results could delay treatment, thus contributing to reservoir maintenance. Recently, several immunological and molecular diagnostic tools have been developed for diagnosis [7,17,19,20]. In particular, the use of molecular techniques has become increasingly relevant due to their high sensitivity, specificity and possible application to a variety of clinical samples. Among them, the real-time quantitative PCR (qPCR) has become increasingly popular recently since it is fast, has a broad dynamic range and cross-contamination is drastically reduced because there is no need to open reaction tubes for post-PCR analyses.

In this paper, we propose a new molecular approach for the diagnosis and study of Kinetoplastida. New TaqMan qPCR assays with different levels of specificity, targeting different genes, have been developed and validated by screening a panel of animal blood samples. The approach was confirmed and completed using PCR/ sequencing technology.

## 2. Material and Methods

### 2.1. Primers and Probe’s Designs

#### 2.1.1. Custom Protocol and In Silico Validation

First, for each PCR assay, the target gene was chosen and a fasta file was constructed from the sequences available in the GenBank database.

Sequences were aligned using BioEdit v 7.0.5.3 software (available online: http://en.bio-soft.net/format/BioEdit.html) [21] to reveal conserved areas suitable as target regions for specific primers and probes. These regions were submitted in Primer3 software v. 0.4.0 (available online: http://primer3.ut.ee/) to determine valuable candidate primers and probes and the selection was based on the criteria for the primer and probe design.

Settings for the PCR primers and probes were in accordance with the guidelines as described by Apte and Daniel [22] and as recommended by Invitrogen™ (available online: https://www.thermofisher.com/fr/fr/home/brands/invitrogen.html) and Applied Biosystems™ (available online: https://www.thermofisher.com/fr/fr/home/brands/applied-biosystems.html). Melting temperatures, secondary structures and the possibility for primer-dimers were tested using the free online software Oligo Analyzer 3.1 (available online: https://eu.idtdna.com/calc/analyzer) [23]. All primer and probe sequences were also checked for their specificity in an NCBI BLAST nucleotide sequence similarity search (available online: https://blast.ncbi.nlm.nih.gov/Blast.cgi) [24]. They were also checked within the DNA databases of metazoans (taxid:33208), vertebrates (taxid:7742), bacteria (taxid:2), *Canidae* (taxid:9608), *Felidae* (taxid:9682) and humans (taxid:9605). This has been done for all possible combinations of forward-reward and probe-reward of each PCR system. Primers were synthesized by Eurogentec (Liège, Belgium) and the hydrolysis probe by Applied Biosystems™. PCR systems and their target genes are described in Table 1.

#### 2.1.2. Specificity-Based Principles of Oligonucleotide Design

**Figure 1:**
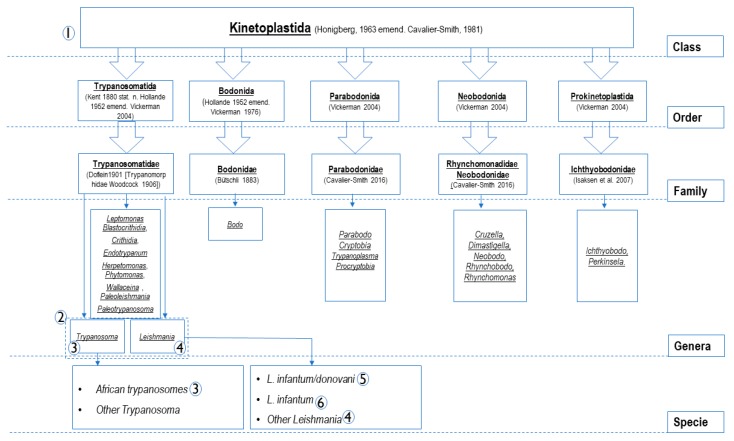
Members belonging to Kinetoplastida class (according to Sina M et al., 2012) [30] and specificity-based principles of the oligonucleotide design. The numbers from 1 to 6 constitute PCRs systems listed in Table 1. As shown in this figure and in the Table 1, PCR systems were designed to amplify the DNA of PCRs (**1**): all Kinetoplastida; PCR (**2**): Leishmania and Trypanosoma spp.; PCRs (**3**): Trypanosoma spp.; PCRs (**4**): Leishmania spp.; PCR (**5**): L. donovani complex (L. donovani/ L. infantum); PCR (**6**): L. infantum.

Unfortunately, no universal primers are disponible for all parasites similar to universal primers FD1/RP2 targeting the *16S rRNA* gene of all bacteria [28]. Studies of the bacterial *16S rRNA* gene have allowed a revolution on phylogeny, taxonomy and several new species, including pathogens, have been highlighted [29]. It seems impossible to find a conserved gene region at the genomic level for all parasites. The following figure (Figure 1) shows members of the Kinetoplastida class [30] and the principles of oligonucleotide design based on specificity in the current approach to study Kinetoplastida of medical and veterinary interest. PCR systems numbered from 1 to 6 (Figure 1 and Table 1) are classed according to their specificity. Target genes are used because of their availability on the database. In addition, generally in eukaryotes, the most conserved and commonly targeted genes for PCR design, are those that encode ribosomal RNA *(rRNA)* [31]. These genes encode small subunit ribosomal *(18S rRNA)* and the two largest subunits ribosomal (*28S* and *5.8S rRNA)* in a transcription unit, constituting about 85–90% of total cellular *RNA*, and are very useful as internal controls [32]. They were the principal targets here especially for PCRs targeting the class, families and genus members. This housekeeping gene has been shown to be optimal in previous studies [33,34]. Further, the conserved region of the *Leishmania* DNA of minicircle kinetoplast *(kDNA)*, recognized for its high sensitivity (several 1000′s-fold repeated sequence) [35], was elected to design qPCR assays for *L. infantum* and *L. donovani*. TaqMan technology was used in this approach. It is a system of light emission that increases the specificity of the reaction. It has several advantages over the classical quantitative PCR system: the use of fluorescent dye-labeled probes increases the sensitivity of the system by at least seven orders of magnitude and gives rise to a linear relationship between the copy number and Ct values [36]. The light emission is proportional to the amount of product generated in the reaction tube. This, in turn, is proportional to the number of initial targets for the amplification reaction [34,37].

### 2.2. Run Protocols

The qPCR reactions were carried out in a final volume of 20 µL, containing 5 µL of DNA template, 10 µL of Master Mix Roche (Eurogentec, Liège, Belgium). In each reaction, we added 0.5 µL of each primer (at 50 µM concentration), 0.5 µL of both UDG and each probe (at 20 µM concentration). Finally, the volume was completed to 20 µL using ultra-purified water DNAse− RNAse free. The TaqMan cycling conditions included two hold steps at 50 °C for 2 min followed by 95 °C for 15 min, and 40 cycles of two steps each (95 °C for 30 s and 60 °C for 30 s). The qPCR amplification was performed in a CFX96 Real-Time system (Bio-rad Laboratories, Foster City, CA, USA) after activating the readers of the dyes (FAM and/or VIC) used in each qPCR system.

### 2.3. Conventional PCR Primer Sets Design, Amplification Protocol and Sequencing

The *28S rRNA* was used to design a primer sets to complete the molecular identification of Kinetoplastids. Primer pair combinations amplify from ~550 to 1300 pb-fragments with a variable specificity among the species belonging to the Kinetoplastida class (Table 1). Primers F0/R2 amplify ~1300 pb. F2/R2 primers can amplify ~920 pb with possibility of hemi-nested PCR after first amplification using primers F0/R2. Primers F2/R1 amplified 550 pb on direct PCR or hemi-nested after first amplification using primers F2/R2. Primers F0/R0 amplify ~800 at 53 °C with possibility of hemi-nested after first amplification using F0/R2. Primers F2/R1 used directly or after first amplification F2/R2, to increase sensitivity, are more suitable.

To identify *Leishmania* spp. a primer pair targeting 550 pb-fragments from the *18S rRNA* gene was used, as well as universal primer pairs targeting *Leishmania* spp. intergenic transcribed spacers 1 and 2 (*ITS1 and ITS2*) [26,38]. In addition, primers ITS1 CF and ITS1 BR [27] targeting *ITS1* partial gene of African trypanosomes were used in this approach.

PCR reactions were performed in a total volume of 50 µL, consisting of 25 µL of AmpliTaq Gold master mix, 18 µL of ultra-purified water DNAse− RNAse free, 1 µL of each primer (at 50 µM concentration) and 5 µL of DNA template. The thermal cycling conditions were incubation step at 95 °C for 15 min, 40 cycles of one minute at 95 °C, 30 s for the annealing at a different melting temperature for each PCR assay (Table 1), 1 min for elongation time at 72 °C followed by a final extension for five minutes at 72 °C. PCR amplification was performed in a Peltier PTC-200 model thermal cycler (MJ Research Inc., Watertown, MA, USA). The results of amplification were visualized by electrophoresis on 2% agarose gel. The purification of PCR products was performed using NucleoFast 96 PCR plates (Macherey Nagel EURL, Hoerdt, France) according to the manufacturer’s instructions. The amplicons were sequenced using a Big Dye Terminator Cycle Sequencing Kit (Perkin Elmer Applied Biosystems, Foster City, CA, USA) with an ABI automated sequencer (Applied Biosystems).

### 2.4. PCR Systems Validation, Specificity, Sensitivity and Efficiency

The assay sensitivity was tested in vitro using, (i) For *Leishmania* PCRs: DNAs of cultivated *Leishmania* species: *L. infantum* MCAN/ES/98/LLM-877 (WHO international reference strain), *L. donovani* (MHOM/IN/00/DEVI), *L. major* (MHOM/IL/81/Friedlin) and DNA of *L. guyanensis* previously detected on red howler monkey from French Guiana (MK782154) [39]; (ii) for *5.8S Trypanosoma* spp. qPCR: DNA panel from cultivated *Trypanosoma* species, *T. congolense IL 3000*, *T. evansi*, *T. vivax*, *T. brucei brucei, T. brucei gambiense* and *T. brucei gambiense biyiamina* group II, *T. cruzi* CL Brenner and DNA of uncultured *T. congolense* detected on a French dog died a few weeks after returning from a 3 months mission in Cote d’Ivoire; (iii) for *28S Leishmania-Trypanosoma* qPCR: All the DNAs cited above; (iv) for *28S* Kinetoplastida based PCRs: All the DNAs cited above, DNA of *Leptomonas* sp. isolated on flea *Ctenocephalides felis* from Senegal and DNA of *Bodo* sp. from the blood of an Algerian dog. All PCR systems were tested for their specificity using several arthropods of laboratory-maintained colonies as well as human, monkey, donkey, horse, cattle, mouse and dog DNAs. DNA collection used to test the sensitivity and specificity of PCR systems are summarized in Table S1.

To assess the analytical sensitivity of the *18S Leishmania* spp. and *kDNA L. donovani/ infantum* based qPCR assays, a standard curve was established using *L. donovani* DNA; 5 µL of serial dilutions ranging from 10,000 to 0.001 parasites were introduced into reaction tubes for each qPCR system. The standard curve concentration was expressed as parasite/mL (par/mL).

Sensitivity of the *28S* Kinetoplastida, *28S Leishmania-Trypanosoma* spp., and the *5.8S Trypanosoma* spp.-based qPCR assays, was assessed using the blood of French dog which died after a *T. congolense* infection, for which the number of parasites was determined by microscopical counting on blood smear (Figure S1). The number of *T. congolense* visualized was 1.06 × 10^6^ parasites/ mL of blood. Twelve-fold dilutions of this DNA (initial concentration of 1.06 × 10^6^ par/ mL) were made for standard curve analysis. In addition, the standard curve concentration was expressed as par/mL. All dilutions were assayed in duplicate. For intra assay validation, two replicates of ten 10-fold DNA concentrations, were assessed in a single assay. Similar dilutions were performed in independent runs for inter assay validation. Variability of the assay is reported as the coefficient of variation (CV) (CV shown as the ratio of mean to standard deviation (SD)) (Table S2). Products of real- time PCR were subjected to gel electrophoresis in 1.5% agarose gel for assessment of analytical specificity.

### 2.5. PCR Tools Validation by Sample Screening and Identification of Kinetoplastida on Biological Samples

An already-existing collection of animal-blood samples was screened by qPCR assays assessed in this study. This includes:

(i) 218 dogs, one horse and one donkey from Kabylia, northern Algeria (May 2018) from the previous study of Medkour et al. 2019 [40].

(ii) 42 dogs from Cote d’Ivoire (April 2018) from the study of Medkour et al. 2020 [25].

(iii) 98 dogs sampled in 2016 and 9 red howler monkeys sampled in 2014 from French Guiana from the previous studies [41] and [39].

The genomic DNA was extracted from 200 µL-blood of each sample after enzymatic digestion with proteinase K, using the QIAGEN DNA tissues extraction kit (QIAGEN, Hilden, Germany), following the manufacturer’s recommendations. The extracted DNA was eluted in a total volume of 200 µL and stored at −20 °C until use.

All samples were screened by all the qPCR and PCR assays (Table 1). Obtained amplicons were then purified and sequenced.

### 2.6. Determination of Assay Performance Characteristics

Here, the performances of qPCR assays were evaluated in the absence of a gold standard test. The true positive samples were determined: The sample was considered true positive if at least one of the sequences was obtained by PCR/sequencing of a part of 28S rRNA for Kinetoplastida, 18S rRNA and/or ITS2 for *Leishmania* spp. and/or ITS1 for *Trypanosoma* spp. (Table 1). Inversely, it was considered false negative when it is negative by qPCR and we obtain sequence after PCR/sequencing. By using this criterion to determine true positive/ negative samples, we reduced the possibility of false positive/ negative and qPCR assays were evaluated as much as possible for their specificities. One of the limitations is the difficulties in taking decisions in cases found positive by qPCR and negative in standard PCR /sequencing that may be linked to a sequencing defect, for unknown reasons, i.e., low DNA quantity in the sample, not related to a defect in the qPCR specificity.

### 2.7. Statistical Analysis

After a set of databases Microsoft Excel^®^ program (Microsoft Corp., Redmont, USA), descriptive analysis of Kinetoplastida infections were performed. The statistical analysis was conducted using XLSTAT Addinsoft version 2018.7 (Data Analysis and Statistical Solution for Microsoft Excel, Paris, France).

To determine assay performance characteristics for each test, prevalence (Pr), correct classification (Cc) and misclassification (Mc), sensitivity (Se), specificity (Sp), false positive rate (FP), false negative rate (FN), positive and negative predictive value (PPV and NPV), were calculated for each test.

To measure the agreement of qPCR assays according to sequencing (defined as gold standard in this study), we could simply calculate the percentage of cases in which qPCR and sequencing results agree. This statistic has nevertheless a significant weakness. It does not account for agreement randomly occurring. On the other hand, Cohen’s Kappa measures agreement, while removing random effects, thus ensuring good reproducibility. Cohen’s Kappa (k) agreement measure was used to evaluate the relevance of each qPCR assay according to the sequencing results as established by Landis and Koch (1977).

## 3. Results

### 3.1. In Silico and In Vitro Validation

In silico validation of the customized PCR and qPCR assays were performed using primer design tools. Assessment of sensitivity and PCR efficiency were confirmed by the equality of melting temperature inside each set of primers, this temperature was less than that of probes. The absence of primer- dimer formation for each system was confirmed.

The specificity was supported by an in vitro validation, qPCR and PCR assays were able to specifically detect target DNAs without failure (Table 2) and no negative control was amplified. Thus, the assays were highly specific for their targets. In addition, no primer dimerization was observed in gel agarose 1.5%.

Artificial *Trypanosoma* and/or *Leishmania* and/or *Leptomonas* DNA mixtures were included to mimic mixed infection controls. It comprised artificially DNA mixed in equal proportions. These controls were processed in the same way as samples until sequencing. The pooled DNAs were successfully detected by qPCR assays (Table 2).

### 3.2. Determining Assay Performance Characteristics: Analytical Sensitivity, Linearity and Reproducibility

The standard curve results spreadsheet (SCRS) of the 28S Kinetoplastida, 28S *Leishmania-Trypanosoma* spp. and the 5.8S *Trypanosoma* spp. based-qPCRs showed: Efficiency (E) of 96.7%, 105.3% and 110.6%; Slope of −3.40, −3.2 and −3.10; as well as an almost perfect correlation coefficient (R^2^) of 0.987, 0.966 and 0.99, respectively. In addition, the limit of detection was fixed at 0.106, 1.06 and 0.0106 par/mL of blood for the three qPCRs respectively (Figures S2–S4).

For the TaqMan 18S *Leishmania* spp. and kDNA *L. donovani* complex based-qPCRs, E was 112.5% and 96.2%, Slope of −3.06 and −3.42, R^2^ was 0.997 and 0.966, and detection limit of 0.1 and 0.01 respectively (Figures S5 and S6).

The intra assay CV of Ct values among the replicates varied homogeneously regardless of quantities of parasite DNAs. The reproducibility of the assay was assessed as an inter assay variation of Ct values for the same dilution series in two independent runs. Inter assay coefficients of variations indicated high reproducibility (Table 3 and Table S2).

### 3.3. Performance Characteristics Comparison of the Diagnostic Tools

Among the 369 samples screened, 45 (12.2%) were positive using the *28S* Kinetoplastida qPCR, 34 samples of them had been identified by sequencing. By using the *28S Leishmania-Trypanosoma* spp. qPCR 10% (37/369) were positive including five samples found negative by the *28S* Kinetoplastida qPCR. Specific qPCR for *Trypanosoma* spp. was able to detect 18/369 (4.9%) as positive including three samples that were negative by both qPCRs cited above. Twenty-one samples (5.7%) were found positive for *Leishmania* spp. and all of them, except one, tested positive in the large screening by the *28S* based-qPCRs. In addition, causative agents of visceral leishmaniasis (VL), *L. infantum/ L. donovani*, were detected in 23/369 (6.2%), including 21/369 samples found positive for *L. infantum*, using the kDNA *L. infantum*- based qPCRs. Four of these samples were found negative using the *18S Leishmania* spp. based-qPCR (Table S3).

Using a method combining sequence typing of the *28S, 18S, ITS1 and ITS2* (Table 1) targeting members of Kinetoplastida parasites 37/369 (10%) of samples were identified to carry at least one pathogen. No sequence was obtained, after PCR/ sequencing, for samples negative by all the qPCR assays. Taking sequencing as the gold standard, our molecular tools (qPCRs) showed a sensitivity ranging from 0.826 to 1, a high specificity from 0.971 to 0.997. A substantial agreement quality with the gold standard test was observed for the 28S *Leishmania*- *Trypanosoma* spp.-based qPCR according to Cohen’s Kappa (Cohen’s Kappa = 0.8). The agreement was almost perfect for all the other qPCR assays (Cohen’s Kappa values from 0.811 to 0.976) (Table 4). The performances were challenged by the presence of false positives (FP), especially for the *28S* based qPCR assays (11 and 8 FP samples for the Kinetoplastida and *Leishmania-Trypanosoma* spp. qPCRs, respectively), and the *Trypanosoma* spp. qPCR (5 FP samples). Greater performances were observed for the *18S* and *kDNA Leishmania*- based qPCR assays (Table 5).

In addition, our PCR tools were able to detect DNA of several Kinetoplastida (*Leishmania infantum*, *Trypanosoma evansi, T. congolense* and *Bodo* spp.) on different hosts (dogs, monkeys, donkeys and horses). It has been also possible to detect co-infections (*L. infantum/T. congolense* con-infection) in two samples (Table S3).

## 4. Discussion

In this study, we propose an approach to explore the presence of different Kinetoplastida parasites in animal or human samples using molecular biology. We have developed and implemented new molecular tools to study these parasites, particularly those of medical and veterinary interest, focusing on visceral leishmaniasis and African trypanosomiasis. Unfortunately, we cannot propose a universal PCR system for all parasites such as the *16S rRNA* PCR system for bacteria. The explosion in the number of recognized taxa and the discovery of new pathogenic bacteria (such: *Bartonella henselae*, *Anaplasma* spp., *Tropheryma whipplei* etc.) is directly attributable to the ease in performance of *16S rRNA* gene sequencing studies [29]. However, our molecular approach allows at least to study Kinetoplastida class. It keeps the doors open to the discovery of pathogens that have not yet been described, especially when it is applicable at a high-level using PCR systems targeting almost all Kinetoplastida members. Furthermore, researchers are free to choose the molecular tools suitable for their studies.

The choice of the target genes for oligonucleotides’ design was based on two principal characteristics: (i) the conserved sequences between targeted species, as is the case with *LSU (28S)* and the *5.8S* genes [42] and their availabilities on databases. Noted that the *24Sα LSU* from the *28S LSU* [43,44] was targeted for the Kinetoplastida class qPCR, a region from the *28S LSU* was targeted for *Leishmania-Trypanosoma* spp. qPCR, *5.8S* gene for *Trypanosoma* spp. qPCR and a conserved region from 18S SSU gene for the pan-*Leishmania* qPCR [45]; (ii) the discrimination between species, as is the case for *kDNA* gene, which was targeted by TaqMan qPCRs for the detection of *L. donovani* complex and the specific detection of *L. infantum* [35]. All the customized PCR assays showed a specific, sensitive and reproducible detection of the targeted DNAs for which they were designed.

Currently, no universal qPCR assay is available for the Kinetoplastida class. A high analytical sensitivity without failure was evaluated for the pan-Kinetoplastida qPCR implemented here. This tool gave an almost perfect agreement (Cohen’s Kappa = 0.81) with the gold standard test defined above. Besides, it allowed us, in addition to *Leishmania*/*Trypanosoma* spp. detection in animal blood samples, to identify *Bodo* spp. (free-living trypanosomes) in the blood of three dogs from Algeria. *Bodo* spp. are rarely detected in blood. One report showed the presence of *Parabodo caudatus* (Kinetoplastida class) in urine voided from a dog with hematuria [46]. The *28S* PCR combination primers were able to distinguish between the Kinetoplastida members at least at the sub-genus level and they allowed discrimination between some species. Other PCR systems pan-Kinetoplastida targeting the *28S LSU* or the *18S SSU* are described [42,47]. Pan-Kinetoplastida PCRs here are suitable for the initial screening especially when there is a large number of samples.

*Leishmania* and *Trypanosoma* genera include the principal pathogenic Kinetoplastids [17,48]. The pan-*Leishmania/Trypanosoma* qPCR was able to detect members of these two genera. It can be used in research for leishmaniasis and trypanosomiasis. The Se and Sp were 0.85 and 0.98 respectively, with a substantial agreement to the gold standard. The weakness of sensitivity could be explained by the detection limit which does not exceed 1 par/mL of blood (Figure S4). Samples found positive in qPCR with no sequence obtained after PCR/sequencing, (Table 5 and Table S1) could be explained by the sequencing defect, for unknown reasons, i.e., low quantity of DNA in the sample, not related to failure in specificity of the qPCRs, especially when some samples were positive in PCR standard but no sequence was found. PCR assays cited later could detect Kinetoplastida’s mixed- infections as single infections, i.e., we cannot decrypt co-infections.

Here, we developed for the first time a pan-*Trypanosoma* qPCR assay highlighted perfect Se, Sp, reproducibility and perfect agreement with the gold standard. It will be useful in epidemiological studies and therapeutic follow-up of African and American trypanosomiasis. The tool was able to detect two additional samples (identified as *T. congolense*) which were negative by both qPCRs targeting the *28S* gene. The cross reactivity between *Trypanosoma* and *Leishmania* spp. is one of the limits for the serological tests, such as ELISA and IFAT [49,50]. No cross-reactivity between trypanosomes and *Leishmania* spp. was detected in system specificity tests compared to other Kinetoplastida (especially *Leishmania* spp.). In addition, the assay is able to detect *Trypanosoma*-genomic DNA in mixed infections with the *Trypanosoma* members or with the other Kinetoplastida (Table 2). Co-infections should be suspected if the *5.8S* qPCR *Trypanosoma* spp. is positive, while sequencing of the *28S* gene for Kinetoplastida is not possible. In this case, genus specific PCR systems (*ITS1* and *ITS2* used for *Trypanosoma* and *Leishmania* spp., respectively, in this study) could decrypt these co-infections. In this study, *T. evansi* and *T. congolense* have been detected in dogs (Table S3).

*Leishmania* spp. qPCR was almost all specific (Sp = 0.994) in the detection of *Leishmania* species. As *Trypanosoma* spp. qPCR, *Leishmania* spp. qPCR overcomes the problem of cross reactivity recognized for serological tests. The tool can be used in studies on animal or human, visceral or cutaneous leishmaniasis, therapeutic follow up by monitoring the parasite burden. *Leishmania* spp. were detected in dogs, monkeys, donkey and horse in the present study. Four samples were negative by this qPCR system where they were positive by the *28S* qPCRs as well as PCR/sequencing (Table S3), therefore the sensitivity did not exceed 0.83. This TaqMan qPCR is able to detect DNA 0.1 par/mL of blood.

Because visceral leishmaniasis is a major problem threating animal and public health, two qPCRs were customized to identify the major causative agents of VL [51]. These tools, together, distinguish between *L. donovani* and *L. infantum* infection since DNA of *L. infantum* has been detected by both assays and *L. donovani* DNA has been detected when used the VIC-TaqMan probe (Table 1). They are suitable for use in endemic area for both *L. infantum* and *L. donovani.* Both assays showed high limit of detection of 0.01 parasites/mL blood and higher Se (1.00) and Sp (0.997) compared to the other qPCR assays. The target gene (*kDNA*) is known for its sensitivity and allows quantification [52,53,54]. The four samples found negative by the *18S* qPCR, were positive by both *kDNA* qPCRs and identified as *L. infantum* previously before going to sequencing.

Based on the results of this study, to applicate this approach, we recommend:

1. For the detection of (practically) all Kinetoplastida parasites an initial screening using at least three qPCRs targeting three different genes for Kinetoplastida, i.e., screening with *28S* Kinetoplastida qPCR with or without screening by the *28S* pan-*Leishmania/Trypanosoma*, followed by screening with the *5.8S* pan-*Trypanosoma* and the *18S* pan-*Leishmania* qPCR assays.
2. *Trypanosoma* spp. and *Leishmania* spp. qPCR assays, in addition to identifying Kinetoplastida at the genus level, they can decrypt co-infections *Leishmania/Trypanosoma*, allow parasite quantification and to define the therapeutic protocol and monitoring (molecules and doses).
3. For the species identification, the *28S* based PCR was able to identify Kinetoplastida, but at the genus or subgenus level. In addition, when there were infections by more than one species, it was not possible to sequence both amplicons.
4. We can resolve this problem using genus-specific PCR systems, as was the case of co-infections by *T. congolense/ L. infantum* in two dogs from Cote d’Ivoire (Table S3). The *kDNA* based qPCRs identified *L. infantum* and *L. donovani* species without sequencing step.

Furthermore, sequencing technology is not widely available in clinical laboratories, hence usefulness of the proposed approach, these labs could conduct studies in Kinetoplastida parasites using only TaqMan qPCR technology.

**Figure 2:**
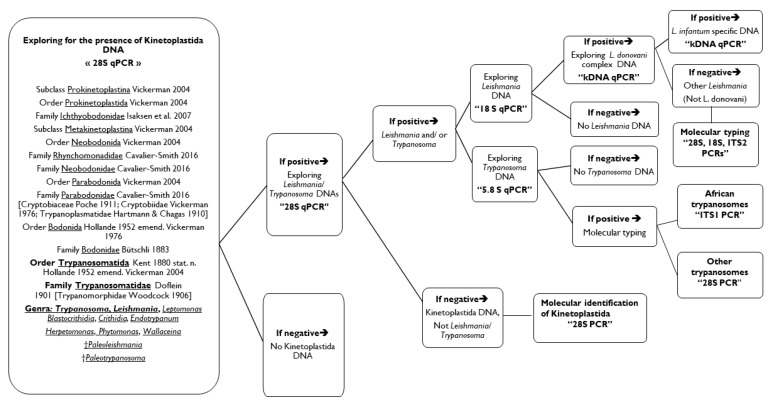
Proposal of the molecular approach for the complete diagnosis of *Kinetoplastida* in human and animal samples.

Finally, we invite researchers to follow the proposed molecular approach (Figure 2) according to their goals. The approach starts at the class level. Samples could be screened for the presence of Kinetoplastida DNA. Positive ones will be tested for *Leishmania*/ trypanosomes DNA. Positives at this stage can be tested for the presence of *Leishmania* DNA on the one hand and for *Trypanosoma* DNA on the other hand. Negatives for *Leishmania* and *Trypanosoma* DNAs will be directly suggested to *28S* standard PCR and sequencing to identify which Kinetoplastid is concerned. Positive samples for *Leishmania* spp. DNA could be tested by the *L. donovani* complex qPCR then by *L. infantum* specific- qPCR. Negatives at this level are necessarily a *Leishmania* sp. other than *L. infantum/ donovani*, and which can be decrypted by genotyping (*28S*, *18S* and/or *ITS2* PCRs). Positives for *Trypanosoma* spp. can be identified using universal primers for African trypanosomes, otherwise by *28S* PCR for all Kinetoplastids.

## 5. Conclusions

The present molecular approach developed here offers researchers the exploration of Kinetoplastida parasites in human and animal samples. It relies principally on TaqMan qPCR and sequencing technologies. Assays were presented, tested and validated. They showed good Se, Sp and reproducibility, with a detection limit of at least a single cell-equivalent of *rDNA*. The approach allows the detection and identification of Kinetoplastida at the genus level for *Trypanosoma* and *Leishmania* spp. two genera including the most important pathogens, by using genus-specific qPCRs. Furthermore, we have implemented a highly sensitive qPCR assay for the detection of visceral leishmaniosis (*L. donovani/infantum*) and a specific qPCR for zoonotic visceral leishmaniosis (*L. infantum*). To identify Kinetoplastids, genotyping was performed by PCR/sequencing for *Leishmania* and *Trypanosoma* spp. A novel primer suite is presented for PCR /sequencing. It allows identification of Kinetoplastida infections detected in animal samples. Besides, this molecular approach is useful in epidemiological studies, in basic research such as probing for new Kinetoplastida species, in diagnosis and therapeutic follow-ups. It could be used in laboratories without sequencing technology.

## Figures and Tables

**Table 1:** Primers and Probes, their Characteristics and Conditions.

PCR Name	Target Gene	Primers and Probes Name	Primers and Probes (5’-3’)	Amplicon Size (pb)	Tm °C	Specificity (Accordance to Figure 1)	Source
qPCR Pan-Kinetoplastida	*28S LSU (24 alpha)*	P. 24a; 5345	FAM-TAGGAAGACCGATAGCGAACAAGTAG	200	60 °C	Kinetoplastida (1)	This study
		F. 24a; 5198	AGTATTGAGCCAAAGAAGG				
		R. 24a; 5412	TTGTCACGACTTCAGGTTCTAT				
qPCR Pan-*Leishmania/ Trypanosoma*	*28S LSU*	P Leish/Tryp	FAM- GGGAAGGATTTCGTGCCAACG	135	60 °C	*Leishmania* spp. and *Trypanosoma* spp. (2)	
		F Leish/Tryp	AGATCTTGGTTGGCGTAG				
		R Leish/Tryp	ATAACGTTGTGCTCAGTTTCC				
qPCR Pan-*Trypanosoma*	*5. 8 S rRNA*	P. 5.8S Tryp	FAM-GTTGAAGAACGCAGCAAAGGCGAT	83	60 °C	*Trypanosoma* spp. (3)	[25]
		F. 5.8S Tryp	CAACGTGTCGCGATGGATGA				
		R. 5.8S Tryp-	ATTCTGCAATTGATACCACTTATC				
qPCR Pan-*Leishmania*	*18S SSU*	P. Leish	FAM- CGGCCGTAACGCCTTTTCAACTCA	75	60 °C	*Leishmania* spp. (4)	
		F. Leish	GGTTTAGTGCGTCCGGTG				
		R. Leish	ACGCCCCAGTACGTTCTCC				
*qPCR L. donovani/ L. infantum*	*kDNA minicircle*	P. *L. inf*	FAM-TGGGCTGGATTGGGTTTTCCTGGGCTGGA	175	60 °C	-VIC: *L. donovani* complex (5) -FAM: *L. infantum* (6)	This study
		P. *L. do* cplx	VIC-TGGGCTCCCCTGGGCTGGATTGGGCTCC				
		F. *L. inf/do*	GGGGTTGGTGTAAAATAGGGCCGGGTGGT				
		R. *L. inf/do*	CCACATCAAAGGCACCCGAACCATTAA				
PCR Pan-Kinetoplastida	*28S LSU*	F2	ACCAAGGAGTCAAACAGACG	(F0/R2) ~1300 (F2/R2) ~920 (F2/R1) ~550	53 °C 53 °C 58 °C	Possibility of hemi-nested PCR: F0/R2 than F2/R2. F2/R1 in direct PCR or hemi-nested after first amplification using F2/R2. Primers F0/R0 amplify ~800 pb at 53 °C with possibility of hemi-nested after amplification by F0/R2. F2/R1 primers more recommended. (1)	
		R1	GACGCCACATATCCCTAAG				
		R2	GTTGGCACGAAATCCTTCC				
		F1	ACCTAGTAGCTGGTTCAC				
		R0	TCAGCATCGCTACAGGCCTC				
PCR Pan-*Leishmania*	*18S SSU*	F1	CTGTGACTAAAGAAGCGTGAC	~550	52 °C	*Leishmania* spp. (4)	
		R1	AGGCCGAATAGAAAAGATACGT				
PCR Pan-*Leishmania*	*ITS 2*	LGITSF2	GCATGCCATATTCTCAGTGTC	370 to 450	60 °C	*Leishmania* spp. (4)	[26]
		LGITSR2	GGCCAACGCGAAGTTGAATTC				
PCR Pan-*Trypanosoma*	*ITS 1*	ITS1-CF	CCGGAAGTTCACCGATATTG	250 to 710	58 °C	African trypanosomes (3)	[27]
		ITS1-BR	TTGCTGCGTTCTTCAACGAA				

**Tm:** Annealing temperature.

**Table 2:** In vitro validation relative specificity of the TaqMan qPCR assays.

qPCR Assay DNA Targets	Kinetoplastida *(28S)*	*Leishmania/Trypanosoma* spp. *(28S)*	*Trypanosoma* spp. *(5.8S)*	*Leishmania* spp. *(18S)*	*L. donovani/L. infantum* *(kDNA)*	*L. infantum* *(kDNA)*
*T. evansi Montecal EC8*	+	+	+			
*T. brucei gambiense biyiamina groupe II*	+	+	+			
*T. brucei*	+	+	+			
*T. brucei gambiense (T. Féo)*	+	+	+			
*T. congolense (Chien Logan)*	+	+	+			
*T. congolense IL 3000*	+	+	+			
*T. congolense (Dog)*	+	+	+			
*T. cruzi CL Brunner*	+	+	+			
*T. cruzi (Dog)*	+	+	+			
*T. vivax*	+	+	+			
*L. infantum*	+	+		+	+	+
*L. donovani*	+	+		+	+	
*L. major*	+	+		+		
*L. guyanensis*	+	+		+		
*Leptomonas* sp.	+					
*Bodo* sp.	+					
*L. infantum + L. donovani*	+	+				
*L. infantum + L. donovani +* *T. congolense IL 3000*	+	+	+			
*T. congolense IL 3000 + T. brucei*	+	+	+			
*T. congolense IL 3000 + T. brucei + Leptomonas* sp.	+	+	+			
**L.:***Leishmania*; **T.:***Trypanosoma*						

**Table 3:** Repeatability and reproducibility of the real-time qPCR assays.

Coefficients of Variation Intra and Inter Assay for qPCR Assays						
	***28S* Kinetoplastida spp.**		***28S Leish/Trypano* pp.**		***5.8S Trypanosoma* spp.**	
***T. Congolense* Load**	**Intra Assay**	**Inter Assay**	**Intra Assay**	**Inter Assay**	**Intra Assay**	**Inter Assay**
1.06 × 10^6^	1.61	2.84	5.13	1.10	1.87	4.43
1.06 × 10^5^	1.88	2.15	4.24	3.04	5.28	7.71
1.06 × 10^4^	2.90	1.82	1.70	0.37	3.44	7.60
1.06 × 10^3^	1.78	9.25	2.72	3.40	2.63	8.80
1.06 × 10^2^	0.85	0.68	2.47	2.66	1.11	4.80
1.06 × 10^1^	3.38	6.97	1.76	1.42	1.99	1.61
1.06 × 10^0^	3.61	9.06	2.12	2.89	0.17	2.05
1.06 × 10^−1^	1.54	9.87	-	-	2.22	3.12
1.06 × 10^−2^	-	1.94	-	-	0.24	1.25
1.06 × 10^−3^	-	-	-	-	-	-
	***18S Leishmania* spp.**		***kDNA L. donovani* cplx**			
*L. Donovani* load	Intra Assay	Inter Assay	Intra Assay	Inter Assay		
1.00 × 10^4^	1.19	1.03	0.94	2.55		
1.00 × 10^3^	1.54	0.42	1.05	0.99		
1.00 × 10^2^	0.32	0.21	0.54	2.90		
1.00 × 10^1^	0.22	2.00	0.41	1.81		
1.00 × 10^0^	1.45	5.74	0.26	0.72		
1.00 × 10^−1^	0.97	1.16	1.47	0.37		
1.00 × 10^−2^	-	-	0.73	0.59		
1.00 × 10^−3^	-	-	-	-		

**Table 4:** Performances of the TaqMan qPCR assays developed in the present study.

Statistic	TaqMan qPCR Systems					
	*28S Kineto*	*28S Leish-Tryp*	*5.8S Tryp*	*18S Leish*	*kDNA L. dono cplx*	*kDNA L. inf*
Correct classification	0.962	0.965	0.986	0.984	0.997	0.998
Misclassification	0.038	0.035	0.014	0.016	0.003	0.002
Sensitivity	0.919	0.853	1.000	0.826	1.000	1.000
Specificity	0.967	0.976	0.986	0.994	0.997	0.997
False positive rate	0.033	0.024	0.014	0.006	0.003	0.003
False negative rate	0.081	0.147	0.000	0.174	0.000	0.000
Prevalence	0.100	0.092	0.035	0.062	0.060	0.051
PPV (Positive predictive value)	0.756	0.784	0.722	0.905	0.957	0.955
NPV (Negative predictive value)	0.991	0.985	1.000	0.989	1.000	1.000
LR+ (Positive likelihood ratio)	27.735	35.717	71.200	142.913	347.000	390.000
LR− (Negative likelihood ratio)	0.084	0.151	0.000	0.175	0.000	0.000
Relative risk	81.600	52.043	-	78.714	-	-
Odds ratio	330.727	237.075	-	817.000	-	-
Cohen’s Kappa	0.81	0.800	0.832	0.855	0.976	0.975
Agreement*	almost perfect	substantial	almost perfect	almost perfect	almost perfect	almost perfect

*Landis and Koch (1977) have established a scale to describe agreement quality according to Kappa values: < 0: no agreement, 0–0.2: small, 0.2–0.4: fair agreement, 0.4–0.6: moderate, 0.6–0.8: substantial, 0.8–1: almost perfect.

**Table 5:** Performances of the TaqMan qPCR assays according to the sequencing results.

TaqMan qPCR Target	Detected and Typed	Detected, Untyped	Typed, Not Detected	Not Detected, Untyped
*28S* Kinetoplastida	34	11	3	321
*28S Leishmania/ Trypanosoma*	29	8	5	327
*5.8S Trypanosoma* spp.	13	5	0	351
*18S Leishmania* spp.	19	2	4	344
*kDNA L. donovani complex*	22	1	0	346
*kDNA L. infantum*	21	1	0	347

## Supplementary Materials

The following are available online at https://www.mdpi.com/2076-2607/8/3/356/s1, Figure S1: *Trypanosoma congolense* in a dog from Cote d’Ivoire; Microscopy G10X100; There were parasite/mL of blood, Figure S2: Standard curve and the detection limit for the TaqMan qPCR *28S LSU* for Kinetoplastida, Figure S3: Standard curve and the detection limit for the TaqMan qPCR *28S LSU Leishmania/ Trypanosoma* spp., Figure S4: Standard curve and the detection limit for the TaqMan qPCR *5.8S rRNA* for *Trypanosoma* spp., Figure S5: Standard curve and the detection limit for the TaqMan qPCR *18S SSU Leishmania* spp., Figure S6: Standard curve and the detection limit for the TaqMan qPCR-*kDNA* for *Leishmania donovani* complex, Table S1: Positive and negative control DNA used for sensitivity and specificity determination of designed oligonucleotides, Table S2: Ct values repeatability and reproducibility of the real time qPCR assays, Table S3: Individual results for samples screened by the qPCR tools and typing.
