# Supplementary material for: New Molecular Approach for the Detection of Kinetoplastida Parasites of Medical and Veterinary Interest
Source: Microorganisms. 2020 Mar 2;8(3):356. doi: 10.3390/microorganisms8030356 (PMC7143920; doi:10.3390/microorganisms8030356)
Supplement: Supplementary file 1 [file microorganisms-08-00356-s001.pdf]

Supplementary Materials

New molecular approach for the detection of Kinetoplastida parasites of medical and veterinary interest

Hacène Medkour [1,2,3], Marie Varloud [4], Bernard Davoust [1,2] and Oleg Mediannikov [1,2,*]

[1] IHU Méditerranée Infection - Microbes, Evolution, Phylogeny and Infection (MEΦI), 13385 Marseille Cedex 05, France; hacenevet1990@yahoo.fr (H.M.); bernard.davoust@gmail.com (B.D.)

[2] UMR Aix-Marseille Université, IRD, APHM -19-21, Bd Jean Moulin, 13385 Marseille Cedex 05, France

[3] PADESCA Laboratory, Veterinary Science Institute, University Constantine 1, 25100, El Khroub, Algeria

[4] CEVA Animal Health, Libourne, France; marie.varloud@ceva.com

[*] Correspondence: olegusss1@gmail.com

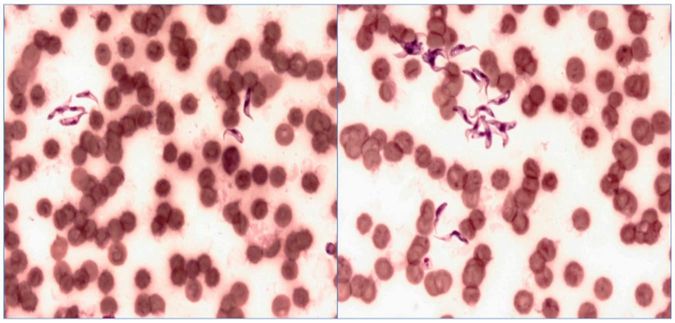

Figure S1: *Trypanosoma congolense* in a dog from Cote d'Ivoire; Microscopy G10X100; There were parasite/mL of blood

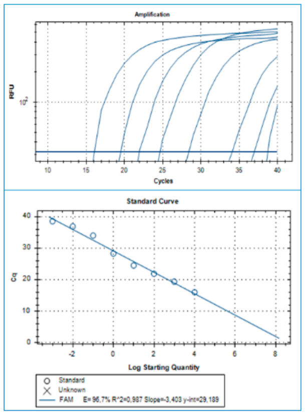

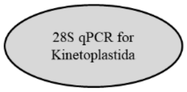

Samples	Content	Cq	SQ
T. congolense	Std 1	16,04	1,06E+06
T. congolense	Std 2	19,41	1,06E+05
T. congolense	Std 3	21,92	1,06E+04
T. congolense	Std 4	24,56	1,06E+03
T. congolense	Std 5	28,34	1,06E+02
T. congolense	Std 6	34,06	1,06E+01
T. congolense	Std 7	36,95	1,06E+00
T. congolense	Std 8	38,61	1,06E-01
T. congolense	Std 9	N/A	1,06E-02
T. congolense	Std 10	N/A	1,06E-03

Figure S2: Standard curve and the detection limit for the TaqMan qPCR 28S LSU for Kinetoplastida

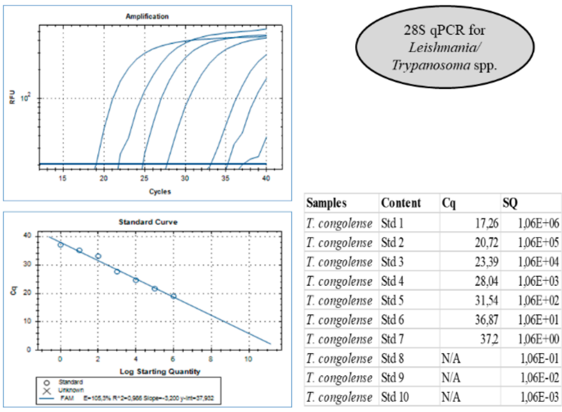

Samples	Content	Cq	SQ
T. congolense	Std 1	17,26	1,06E+06
T. congolense	Std 2	20,72	1,06E+05
T. congolense	Std 3	23,39	1,06E+04
T. congolense	Std 4	28,04	1,06E+03
T. congolense	Std 5	31,54	1,06E+02
T. congolense	Std 6	36,87	1,06E+01
T. congolense	Std 7	37,2	1,06E+00
T. congolense	Std 8	N/A	1,06E-01
T. congolense	Std 9	N/A	1,06E-02
T. congolense	Std 10	N/A	1,06E-03

Figure S3: Standard curve and the detection limit for the TaqMan qPCR 28S LSU *Leishmania/ Trypanosoma spp*.

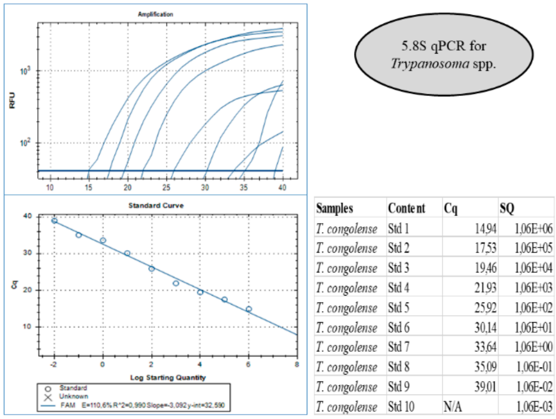

Samples	Content	Cq	SQ
T. congolense	Std 1	14,94	1,06E+06
T. congolense	Std 2	17,53	1,06E+05
T. congolense	Std 3	19,46	1,06E+04
T. congolense	Std 4	21,93	1,06E+03
T. congolense	Std 5	25,92	1,06E+02
T. congolense	Std 6	30,14	1,06E+01
T. congolense	Std 7	33,64	1,06E+00
T. congolense	Std 8	35,09	1,06E-01
T. congolense	Std 9	39,01	1,06E-02
T. congolense	Std 10	N/A	1,06E-03

Figure S4: Standard curve and the detection limit for the TaqMan qPCR 5.8S rRNA for *Trypanosoma* spp.

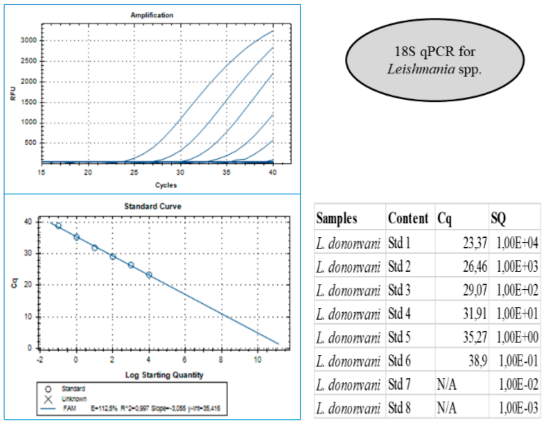

Samples	Content	Cq	SQ
L. dononvani	Std 1	23,37	1,00E+04
L. dononvani	Std 2	26,46	1,00E+03
L. dononvani	Std 3	29,07	1,00E+02
L. dononvani	Std 4	31,91	1,00E+01
L. dononvani	Std 5	35,27	1,00E+00
L. dononvani	Std 6	38,9	1,00E-01
L. dononvani	Std 7	N/A	1,00E-02
L. dononvani	Std 8	N/A	1,00E-03

Figure S5: Standard curve and the detection limit for the TaqMan qPCR 18S SSU *Leishmania* spp.

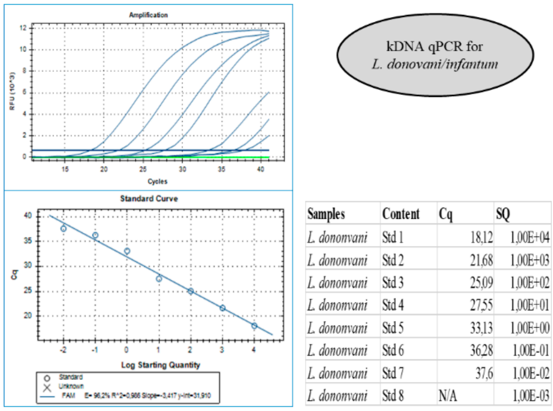

Samples	Content	Cq	SQ
L. dononvani	Std 1	18,12	1,00E+04
L. dononvani	Std 2	21,68	1,00E+03
L. dononvani	Std 3	25,09	1,00E+02
L. dononvani	Std 4	27,55	1,00E+01
L. dononvani	Std 5	33,13	1,00E+00
L. dononvani	Std 6	36,28	1,00E-01
L. dononvani	Std 7	37,6	1,00E-02
L. dononvani	Std 8	N/A	1,00E-03

Figure S6: Standard curve and the detection limit for the TaqMan qPCR-kDNA for *Leishmania donovani* complex

Table S1. Positive (*) and negative control DNA used for sensitivity and specificity determination of designed oligonucleotides

N°	Organism	Name
1	Protozoa	*Leishmania infantum**
2	Protozoa	*Leishmania major**
3	Protozoa	*Leishmania donovani**
4	Protozoa	*Leishmania guyanensis**
5	Protozoa	*Trypanosoma evansi**
7	Protozoa	*Trypanosoma brucei brucei**
8	Protozoa	*Trypanosoma brucei gambiense*
9	Protozoa	*Trypanosoma brucei gambiense* biyiamina groupe II*
10	Protozoa	*Trypanosoma congolense* IL 3000*
12	Protozoa	*Trypanosoma vivax* *
14	Protozoa	*Trypanosoma cruzi CL Brunner**

15	Protozoa	*Trypanosoma cruzi (Dog KIM)**
16	Protozoa	*Leptomonas* spp. *
17	Bacteria	*Rickettsia montanensis*
18	Lice	Head lice *(Pediculus humanus capitis)*
19	Bacteria	*Ehrlichia canis*
20	Bacteria	*Coxiella burnetii*
21	Bacteria	*Borrelia recurrentis*
22	Bacteria	*Staphylococcus hominis*
23	Bacteria	*Asaia bogorensis*
24	Piroplasm	*Babesia canis (*Moocky*)*
25	Bacteria	*Haemophilus influenzae*
27	Bacteria	*Asaia* C2
28	Bacteria	*Wolbachia* sp.
29	Bacteria	*Anaplasma phagocytophilum*
30	Bacteria	*Enterobacter aerogenes*
31	Bacteria	*Streptococcus pneumoniae*
32	Bacteria	*Salmonella enterica*
33	Bacteria	*Citrobacter koseri*
34	Bacteria	*Gardnerella vaginalis*
35	Bacteria	*Streptococcus pyogenes*
36	Protozoa	*Plasmodium* sp.
37	Bacteria	*Rickettsia typhi*
38	Bacteria	*Enterococcus faecium*
39	Bacteria	*Streptococcus agalactiae*
40	Bacteria	*Rickettsia conorii*
41	Tick	*Rhipicephalus microplus*
42	Bovine	BA286 cell line
43	Dog	DH62 cell line
44	Human	HL60 cell line *(Homo sapiens)*
45	Donkey	ANE 4 *(Equus asinus)*
46	Horse	CV.G 22 *(Equs caballus)*
47	Flea	*Ctenocephalides felis*
48	Bedbugs	*Cimex lectularius*
49	Tick	*Hyalomma marginatum*
50	Tick	*Amblyomma variegatum*
51	Dog	DH62 dog cell line (*Canis lupus familiaris*)
52	Protozoa	*Hepatozoon canis*
53	Protozoa	*Leptomonas saimouri*
54	Piroplasm	*Theileria equi*
55	Bacteria	*Rickettsia massiliae*
56	Bacteria	*Staphylococcus haemolyticus*
57	Bacteria	*Staphylococcus aureus*
58	Bacteria	*Rickettsia felis*
59	Bacteria	*Stenotrophomonas maltophilia*
60	Bacteria	*Acinetobacter sp.*
61	Bacteria	*Enterobacter aerogenes*

Table S2. Ct values repeatability and reproducibility of the real time qPCR assays.

kDNA *Leishmania donovani* complex qPCR

	Intra assay variations of Ct values					Inter assay variations of Ct values				
***L. donovani* load**	**Replicate 1**	**Replicate 2**	**Mean**	**SD**	**CV %**	**Assay 1**	**Assay 2**	**Mean**	**SD**	**CV %**
1,00E+04	18,12	17,88	18	0,17	0,94	18	18,66	18,33	0,47	2,55
1,00E+03	21,68	21,36	21,52	0,23	1,05	21,52	21,22	21,37	0,21	0,99
1,00E+02	25,09	24,9	24,995	0,13	0,54	24,995	23,99	24,49	0,71	2,90
1,00E+01	27,55	27,71	27,63	0,11	0,41	27,63	26,93	27,28	0,49	1,81
1,00E+00	33,13	33,01	33,07	0,08	0,26	33,07	33,41	33,24	0,24	0,72
1,00E-01	36,28	37,04	36,66	0,54	1,47	36,66	36,47	36,57	0,13	0,37

1,00E-02	37,6	37,99	37,795	0,28	0,73	37,795	38,11	37,95	0,22	0,59
1,00E-03	-	-	-	-	-	-	-	-	-	-

18S *Leishmania* spp. qPCR

	Intra assay variations of Ct values					Inter assay variations of Ct values				
***L. donovani* load**	**Replicate 1**	**Replicate 2**	**Mean**	**SD**	**CV %**	**Assay 1**	**Assay 2**	**Mean**	**SD**	**CV %**
1,00E+04	23,37	22,98	23,175	0,28	1,19	23,175	22,84	23,01	0,24	1,03
1,00E+03	26,46	25,89	26,175	0,40	1,54	26,175	26,33	26,25	0,11	0,42
1,00E+02	29,07	28,94	29,005	0,09	0,32	29,005	29,09	29,05	0,06	0,21
1,00E+01	31,91	32,01	31,96	0,07	0,22	31,96	31,07	31,52	0,63	2,00
1,00E+00	35,27	36	35,635	0,52	1,45	35,635	38,65	37,14	2,13	5,74
1,00E-01	38,9	39,44	39,17	0,38	0,97	39,17	39,82	39,50	0,46	1,16
1,00E-02	-	-	-	-	-	-	-	-	-	-
1,00E-03	-	-	-	-	-	-	-	-	-	-

5.8S *Trypanosoma* spp. qPCR

	Intra assay variations of Ct values					Inter assay variations of Ct values				
***T. congolense*load**	**Replicate 1**	**Replicate 2**	**Mean**	**SD**	**CV %**	**Assay 1**	**Assay 2**	**Mean**	**SD**	**CV %**
1,06E+06	14,94	15,34	15,14	0,28	1,87	15,14	16,12	15,63	0,69	4,43
1,06E+05	17,53	18,89	18,21	0,96	5,28	18,21	20,31	19,26	1,48	7,71
1,06E+04	19,46	20,43	19,945	0,69	3,44	19,945	22,21	21,08	1,60	7,60
1,06E+03	21,93	22,76	22,345	0,59	2,63	22,345	25,31	23,83	2,10	8,80
1,06E+02	25,92	26,33	26,125	0,29	1,11	26,125	27,96	27,04	1,30	4,80
1,06E+01	30,14	31	30,57	0,61	1,99	30,57	29,88	30,23	0,49	1,61
1,06E+00	33,64	3,36E+01	33,6	0,06	0,17	33,6	32,64	33,12	0,68	2,05
1,06E-01	35,09	36,21	35,65	0,79	2,22	35,65	34,11	34,88	1,09	3,12
1,06E-02	39,01	39,14	39,075	0,09	0,24	39,075	38,39	38,73	0,48	1,25
1,06E-03	-	-	-	-	-	-	-	-	-	-

28S *Leishmania/Trypanosoma* spp. qPCR

	Intra assay variations of Ct values					Inter assay variations of Ct values				
***T. congolense*load**	**Replicate 1**	**Replicate 2**	**Mean**	**SD**	**CV %**	**Assay 1**	**Assay 2**	**Mean**	**SD**	**CV %**
1,06E+06	17,26	18,56	17,91	0,92	5,13	17,91	18,19	18,05	0,20	1,10
1,06E+05	20,72	22	21,36	0,91	4,24	21,36	22,3	21,83	0,66	3,04
1,06E+04	23,39	23,96	23,675	0,40	1,70	23,675	23,55	23,61	0,09	0,37
1,06E+03	28,04	29,14	28,59	0,78	2,72	28,59	30	29,30	1,00	3,40
1,06E+02	31,54	32,66	32,1	0,79	2,47	32,1	33,33	32,72	0,87	2,66
1,06E+01	36,87	37,8	37,335	0,66	1,76	37,335	38,09	37,71	0,53	1,42
1,06E+00	37,2	38,33	37,765	0,80	2,12	37,765	39,34	38,55	1,11	2,89
1,06E-01	-	-	-	-	-	-	-	-	-	-
1,06E-02	-	-	-	-	-	-	-	-	-	-
1,06E-03	-	-	-	-	-	-	-	-	-	-

28S Kinetoplastida spp. qPCR

	Intra assay variations of Ct values					Inter assay variations of Ct values				
***T. congolense*load**	**Replicate 1**	**Replicate 2**	**Mean**	**SD**	**CV %**	**Assay 1**	**Assay 2**	**Mean**	**SD**	**CV %**
1,06E+06	16,04	15,68	15,86	0,25	1,61	15,86	16,51	16,19	0,46	2,84
1,06E+05	19,41	18,9	19,155	0,36	1,88	19,155	18,58	18,87	0,41	2,15

1,06E+04	21,92	21,04	21,48	0,62	2,90	21,48	22,04	21,76	0,40	1,82
1,06E+03	24,56	23,95	24,255	0,43	1,78	24,255	27,65	25,95	2,40	9,25
1,06E+02	28,34	28	28,17	0,24	0,85	28,17	27,9	28,04	0,19	0,68
1,06E+01	34,06	32,47	33,265	1,12	3,38	33,265	30,14	31,70	2,21	6,97
1,06E+00	36,95	35,11	36,03	1,30	3,61	36,03	31,69	33,86	3,07	9,06
1,06E-01	38,61	37,78	38,195	0,59	1,54	38,195	33,21	35,70	3,52	9,87
1,06E-02		39,5	39,5	-	-	39,5	38,43	38,97	0,76	1,94
1,06E-03	-	-	-	-	-	-	-	-	-	-

Table S3. Individual results for samples screened by the qPCR tools and typing.

Sample's ID	Species	Country	28 S Kineto	28 S Leish-Tryp	5.8S Tryp	18S Leish	kDNA L. dono Cplx	kDNA L. inf	Typing
1	Dog	Algeria	+	+	-	+	+	+	*L. infantum*
2	Dog	Algeria	+	+	-	-	-	-	-
3	Dog	Algeria	+	-	-	-	-	-	-
3	Dog	Algeria	+	-	-	-	-	-	*B. caudatus*
4	Dog	Algeria	+	-	-	-	-	-	*B. caudatus*
5	Dog	Algeria	+	-	-	-	-	-	*B. caudatus*
6	Dog	Algeria	+	-	-	-	-	-	-
7	Dog	Algeria	-	+	-	-	-	-	-
8	Dog	Algeria	+	-	-	-	-	-	-
9	Dog	Algeria	+	-	-	-	-	-	-
10	Dog	Algeria	+	+	-	-	-	-	-
11	Dog	Algeria	-	+	-	+	+	+	*L. infantum*
12	Dog	Algeria	+	+	-	-	+	+	*L. infantum*
13	Dog	Algeria	+	-	-	-	+	+	*L. infantum*
14	Dog	Algeria	+	+	+	-	-	-	*T. evansi*
15	Dog	Algeria	-	+	-	-	-	-	-
16	Dog	Algeria	+	+	+	-	-	-	*T. evansi*
17	Dog	Algeria	+	+	+	-	-	-	*T. evansi*
S1	Dog	Algeria	+	-	-	-	-	-	-
S11	Dog	Algeria	+	-	-	+	+	+	*L. infantum*
S14	Dog	Algeria	+	+	+	-	-	-	*T. congolense*
S17	Dog	Algeria	+	+	+	-	-	-	*T. congolense*
S26	Dog	Algeria	+	+	-	+	+	-	*L. donovani/ L. infantum*
S27	Dog	Algeria	+	+	-	+	+	+	*L. donovani/L. infantum*
S33	Dog	Algeria	+	+	-	+	+	+	*L. infantum*
S41	Dog	Algeria	+	+	+	-	-	-	*T. evansi*
S49	Dog	Algeria	+	+	+	-	-	-	*T. evansi*
S50	Dog	Algeria	+	+	-	+	+	+	*L. infantum*
S54	Dog	Algeria	+	-	-	-	+	+	*L. infantum*
S73	Dog	Algeria	+	+	-	-	-	-	No sequence
B5	Dog	Algeria	+	+	+	-	-	-	*T. evansi*
B6	Dog	Algeria	+	+	+	-	-	-	*T. evansi*
T3	Dog	Algeria	+	+	-	+	+	+	*L. donovani/L. infantum*
ANE	Equid	Algeria	+	+	-	+	+	+	*L. infantum*
CHEVAL	Equid	Algeria	+	+	-	+	+	+	*L. infantum*
CMT 21	Dog	French Guiana	+	+	-	+	+	+	*L. infantum*
CMT 72	Dog	French Guiana	+	-	-	-	-	-	-
CMT 78	Dog	French Guiana	-	+	+	-	-	-	No sequence
CMT 80	Dog	French Guiana	+	+	-	+	+	+	*L. infantum*
CMT 86	Dog	French Guiana	-	+	+	-	-	-	No sequence
CMT 89	Dog	French Guiana	-	-	+	-	-	-	No sequence
CMT 94	Dog	French Guiana	+	-	-	-	-	-	No sequence
CMT 95	Dog	French Guiana	+	+	+	+	+	+	*L. infantum*
B2	Monkey	French Guiana	+	+	-	+	+	+	*L. infantum*
B6	Monkey	French Guiana	+	+	-	+	+	+	*L. infantum*

**B8**	Monkey	French Guiana	+	+	-	+	-	-	*L. guyanensis*
B9	Monkey	French Guiana	-	-	-	+	-	-	-
**EA16**	Dog	Cote d'Ivoire	**-**	**-**	**+**	-	-	-	*T. congolense*
**EA33**	Dog	Cote d'Ivoire	+	+	+	**-**	**+**	**+**	*T. congolense/ L. infantum*
**EA40**	Dog	Cote d'Ivoire	+	+	+	+	+	-	-
**EA56**	Dog	Cote d'Ivoire	**-**	**-**	**+**	-	-	-	*T. congolense*
**EA64**	Dog	Cote d'Ivoire	+	+	+	+	+	+	*T. congolense/ L. infantum*
**EA79**	Dog	Cote d'Ivoire	+	+	-	+	+	+	*L. infantum*
**EA95**	Dog	Cote d'Ivoire	+	+	-	+	+	+	*L. infantum*

No sequence: means PCR + but not sequence obtained
